# Targeting the Hepcidin-Ferroportin Axis in the Diagnosis and Treatment of Anemias

**DOI:** 10.1155/2010/750643

**Published:** 2009-12-24

**Authors:** Elizabeta Nemeth

**Affiliations:** David Geffen School of Medicine at UCLA, CHS 37-131, 10833 LeConte Avenue, Los Angeles, CA 90095-1690, USA

## Abstract

The hepatic peptide hormone hepcidin regulates dietary iron absorption, plasma iron concentrations, and tissue iron distribution. Hepcidin acts by causing the degradation of its receptor, the cellular iron exporter ferroportin. The loss of ferroportin decreases iron flow into plasma from absorptive enterocytes, from macrophages that recycle the iron of senescent erythrocytes, and from hepatocytes that store iron, thereby lowering plasma iron concentrations. Malfunctions of the hepcidin-ferroportin axis contribute to the pathogenesis of different anemias. Deficient production of hepcidin causes systemic iron overload in iron-loading anemias such as beta-thalassemia; whereas hepcidin excess contributes to the development of anemia in inflammatory disorders and chronic kidney disease, and may cause erythropoietin resistance. The diagnosis of different forms of anemia will be facilitated by improved hepcidin assays, and the treatment will be enhanced by the development of hepcidin agonists and antagonists.

## 1. Hepcidin-Ferroportin Interaction Regulates Iron Homeostasis

Hepcidin is a small peptide hormone secreted by hepatocytes, circulating in blood plasma and excreted in urine [1]. Like other peptide hormones, hepcidin is synthesized initially as a larger 84-amino acid preprohepcidin then processed in hepatocytes by the signal peptidase to 60-amino acid prohepcidin that lacks iron-regulatory activity [2]. Prior to secretion, prohormone convertases cleave prohepcidin at a polybasic motif to generate the mature bioactive 25-amino acid hepcidin [3]. Other cell types including macrophages and adipocytes also contain hepcidin mRNA but their local and systemic contribution to the production of bioactive hepcidin has not been established with certainty. Hepcidin plays an essential role in maintaining iron homeostasis, and the dysregulation of its production underlies many iron disorders. Chronic excess of hepcidin causes iron-restricted anemia [4], whereas hepcidin deficiency results in iron overload with iron deposition in the liver and other parenchyma [5]. 

**Figure 1:**
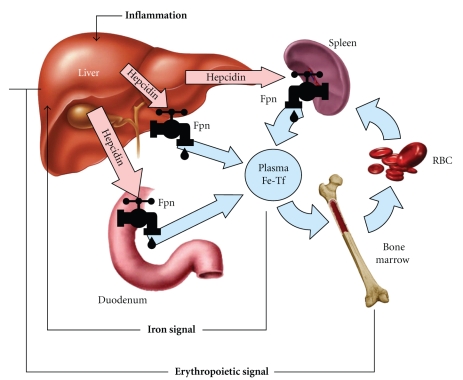
Hepcidin-ferroportin interaction determines the flow of iron into plasma. Hepcidin concentration is in turn regulated by iron, erythropoietic activity, and inflammation.

Hepcidin acts by regulating the cellular concentration of its receptor, ferroportin. Ferroportin is the sole known cellular iron exporter and is essential for iron homeostasis [6]. This multispanning membrane protein is expressed in tissues which transport large amounts of iron (Figure 1): duodenal enterocytes which absorb dietary iron, macrophages of the spleen and liver which recycle iron from old erythrocytes, hepatocytes which store and release iron according to body needs, and placental trophoblast which transports iron from maternal to fetal circulation [7–9]. 

When ferroportin is located in the cell membrane it allows efflux of iron from the cells into plasma. Hepcidin binding to the extracellular face of ferroportin triggers internalization and degradation of the ligand-receptor complex [10]. Removal of ferroportin from the membrane stops cellular iron export leading to decreased supply of iron into plasma (Figure 1). Without the constant iron influx, the plasma iron pool is rapidly depleted by the iron-consuming cells, most prominently erythroid precursors. In mice, a single injection of synthetic hepcidin caused a rapid drop in serum iron [11], and this lasted for 2 days, presumably until sufficient amount of ferroportin was resynthesized. Decreased ferroportin concentration in cell membranes, as seen during chronic overproduction of hepcidin, can lead to iron-restricted erythropoiesis. Interestingly, ferroportin is also expressed in erythroid precursor cells [12], but its physiological role or the effect of hepcidin on developing erythrocytes remains to be determined. 

## 2. Regulation of Hepcidin

Hepcidin is homeostatically regulated by iron and erythropoietic activity. Increased plasma and stored iron stimulate hepcidin production, which in turn blocks dietary iron absorption and further iron loading (Figure 1). Hepcidin is suppressed in iron deficiency [13], allowing increased absorption of dietary iron and replenishment of iron stores. The feedback loop between iron and hepcidin ensures the stability of plasma iron concentrations.

As would be expected for the iron-regulatory hormone, hepcidin production is also regulated by the process which consumes most iron, erythropoiesis [14]. Increased erythropoietic activity suppresses hepcidin production which allows the release of stored iron from macrophages and hepatocytes, and increased iron absorption, all resulting in greater supply of iron for hemoglobin synthesis. 

Hepcidin production is also pathologically increased in inflammation and infection [15]. Resultant hypoferremia may represent a host defense strategy to limit iron availability to microorganisms, but can also lead to iron dysregulation and iron-restricted anemia in inflammatory diseases.

## 3. Molecular Mechanisms of Hepcidin Regulation

### 3.1. Iron

Hepcidin is likely regulated by both circulating iron-transferrin and intracellular iron stores. Although the respective mechanisms of sensing extracellular and intracellular iron are not well understood, they both appear to utilize the bone morphogenetic protein (BMP) pathway to alter hepcidin expression. Several BMPs have been shown to increase hepcidin production in vitro and in vivo [16], but BMP6 has recently emerged as the principal endogenous BMP regulating hepcidin. BMP6 knockout mice develop severe iron overload but no other significant abnormalities [17, 18]. 

In other biological settings, BMP signaling is known to be modulated by coreceptors and antagonists. Hemojuvelin (HJV), a GPI-linked membrane protein, appears to be the co-receptor specialized for iron regulation [19]. The soluble form of hemojuvelin acts as an antagonist, probably by binding BMPs, but the biological role of this interaction has not been documented yet [20]. HJV mutations in humans or mice result in severe iron overload similar to that caused by ablation of hepcidin, without any other apparent problems [21]. Additional molecules, including a protease TMPRSS6 [22] and a large multifunctional transmembrane protein neogenin [23], were shown to interact with HJV and modify its cell-surface expression. Whether these molecules or their interaction with HJV is modulated by iron or other signals remains to be determined.

The mechanism by which intracellular iron regulates hepcidin expression is still unclear. However, expression of BMP6 mRNA was recently shown to increase with iron loading in mice, raising the possibility that BMP6 is a signal reflecting iron stores [24]. Hepcidin regulation by extracellular iron is better understood, and a tentative model is emerging but needs further experimental support. Sensing of iron-transferrin concentrations apparently depends on transferrin receptor 2 (TfR2) and HFE, two molecules which are mutated in the adult form of hereditary hemochromatosis. TfR2 is a homolog of TfR1, but is primarily expressed in the liver, the site of hepcidin expression. Binding of iron-transferrin to TfR2 stabilizes the protein [25], which results in elevated ERK1/2 and Smad1/5/8 signaling [26]. HFE, a protein similar to MHC I type molecules, appears to function as a shuttle between TfR1 and TfR2 depending on iron-transferrin concentrations [27]. Because the binding sites of HFE and iron-transferrin on TfR1 overlap, higher iron-transferrin concentrations displace HFE from TfR1 allowing HFE to associate with TfR2, which presumably potentiates signaling pathways downstream of TfR2. HFE and TfR2, however, do not appear to be required for hepcidin regulation by iron stores, as mice and humans with HFE and TfR2 mutations are still capable of decreasing hepcidin levels after iron depletion [28].

### 3.2. Erythropoiesis

Increased erythropoietic activity is a potent suppressor of hepcidin production. A single injection of erythropoietin in humans caused a dramatic decrease in serum hepcidin within 24 hours [29], and a mouse model showed a dose-dependent decrease in hepcidin mRNA after erythropoietin administration [30]. Epo by itself does not appear to be a direct regulator of hepcidin expression because pretreatment of mice with carboplatin, a cytotoxic inhibitor of erythropoiesis, completely abrogated the effect of Epo on hepcidin [14]. Similarly, mouse models of anemias caused by bleeding or hemolysis showed that hepcidin suppression depended on intact erythropoietic activity [14, 31].

How erythropoiesis affects hepcidin production is not clear, but the mediators could include the production of soluble factors by the erythroid precursors in the bone marrow, decreased circulating or stored iron, and hypoxia. Two proteins produced by erythroid precursors, growth differentiation factor 15 (GDF15) and twisted gastrulation protein (TWSG1), have been proposed to mediate hepcidin suppression in anemias with ineffective erythropoiesis [32, 33]. GDF15, a member of the TGF-*β* superfamily, and TWSG1, a BMP-binding protein, are both produced by developing erythroblasts. The two proteins were shown to suppress hepcidin mRNA in vitro [32, 33]. Very high levels of GDF15 were detected in *β*-thalassemia and congenital dyserythropoietic anemia type I, and elevated Twsg1 expression was found in a mouse model of thalassemia. However, physiologic hepcidin suppression in response to bleeding or to anemias with effective erythropoiesis is likely mediated by other mechanisms. Whatever the signal, erythroid activity appears to suppress hepcidin, at least in part, by modulating the BMP pathway. Erythropoietin administration in mice, which caused the expected decrease in hepcidin levels, was also found to reduce Smad signaling [30].

The physiological relevance of hepcidin regulation by hypoxia is still uncertain. Alterations of the HIF pathway in vivo can affect hepcidin expression [34] but whether HIF regulates hepcidin transcription directly or mostly indirectly is still unresolved. It is possible that the main effect of hypoxia on iron homeostasis is to increase erythropoietin production in the kidney, which would lead to proliferation of erythroblasts and suppression of hepcidin by putative erythroid factors.

### 3.3. Inflammation

Hepcidin synthesis is rapidly increased by infection and inflammation, causing retention of iron in macrophages and decreased iron absorption [15]. The resulting hypoferremia is presumably a component of innate immune responses that deprive invading microbes of iron and other essential nutrients. Serum hepcidin was found to be greatly increased in patients with inflammation defined as CRP >10 mg/dL, patients with sepsis, burns, inflammatory bowel disease, and multiple myeloma [13, 35–37]. Of inflammatory mediators regulating hepcidin, IL-6 was shown to be a prominent inducer in vitro and in vivo, and it stimulates hepcidin transcription through a STAT-3 dependent mechanism [38, 39]. Other cytokines such as IL-1 may also directly regulate hepcidin production, at least in mice, as IL-6 knockout mice with chronic inflammation increased hepcidin mRNA similarly to wild-type mice [40]. Direct regulation of hepcidin synthesis in myeloid cells by microbial molecules acting through toll-like receptors has also been proposed [41] but it is not clear how much this mechanism contributes to hepcidin production locally or systemically, and whether it also applies to hepatocytes.

## 4. The Role of Hepcidin in Anemias

Hepcidin expression is the result of the interplay between multiple stimuli, including iron, inflammation, and erythropoiesis. Accordingly, hepcidin concentrations in different forms of anemia vary widely, and may have diagnostic potential in differentiating between the various types of anemia. Furthermore, in cases where hepcidin is a causative factor in anemia, hepcidin-targeted therapies may improve treatment options for the patients.

### 4.1. Iron Deficiency Anemia (IDA)

In pure iron deficiency anemia, serum and urinary hepcidin concentrations are greatly decreased and are frequently undetectable by currently available assays [13]. The low expression is presumably due to the lack of transcriptional stimulation by iron as well as active suppression by erythroid factors. Hepcidin appears to be a sensitive indicator of iron deficiency even in the absence of anemia. Decreased hepcidin, together with low transferrin saturation and serum ferritin, is observed prior to a detectable decrease in Hb or Hct ([13], E. Nemeth unpublished). Hepcidin measurements could improve the screening of blood donors, in whom deferral is currently based on low Hct or Hb levels, the relatively late sequelae of iron deficiency. Identifying donors who are already iron-deficient prior to the blood donation should reduce the frequency of frank iron deficiency and anemia in blood donors.

Hepcidin is readily detectable in urine [13] and thus may be an excellent candidate for the development of a simple field test for iron deficiency. Apart from the possible use for easy screening of infants and small children, a field-friendly test would be particularly useful in areas which lack access to clinical laboratories. Accurate detection of iron deficiency has become an important issue in endemic malarial regions. “The Pemba trial,” a randomized controlled trial in Zanzibar [42] which evaluated the impact of iron, folic acid, and zinc supplementation on morbidity and mortality in young children, found an increased risk of severe illness and death in children supplemented with iron/folic acid. Furthermore, the risk appeared to be restricted to iron-replete children. Thus, a simple, quick hepcidin test would help distinguish between iron-deficient/anemic children who can benefit from iron supplementation, and iron-replete or already infected children in whom iron supplementation might be harmful. 

### 4.2. Iron-Refractory Iron Deficiency Anemia (IRIDA)

Iron-refractory iron deficiency anemia is a hereditary hypochromic, microcytic anemia, refractory to treatment with oral iron, and only partially responsive to parenteral iron. The molecular basis of the disease was only recently described. IRIDA is caused by increased hepcidin production due to mutations in the hepcidin suppressor, TMPRSS6 also called matriptase-2 [43, 44]. The disease is thus a phenotypic opposite of juvenile hemochromatosis caused by disruptive hepcidin mutations. TMPRSS6 is a membrane protease which was reported to act by cleaving membrane-associated HJV [22], which presumably results in decreased BMP pathway activity. When TMPRSS6 is mutated, hepcidin expression is increased, chronically inhibiting iron absorption and resulting in the development of iron deficiency anemia. How TMPRSS6 expression and activity are regulated in relation to iron homeostasis still remains to be determined. 

Interestingly, TMPRSS6 locus is proving to be highly polymorphic. If some common polymorphisms confer changes in TMPRSS6 expression or activity, this could in turn affect hepcidin expression and the control of iron metabolism. Recently, genome-wide association studies identified association between certain TMPRSS6 variants and serum iron, transferrin saturation, MCV and hemoglobin levels [45, 46]. These findings suggest that even subtle variants in hepcidin regulators have an influence on iron status, erythropoiesis, and associated disorders in the general population. Improved definition of genetic risk factors for iron deficiency or iron overload could help avoid unnecessary burdens associated with chronic treatment.

### 4.3. Iron-Refractory Anemia Associated with Hepcidin-producing Tumors

Another rare form of iron-refractory iron deficiency anemia is associated with hepatic adenomas. Originally reported in the setting of type 1a glycogen storage disease, patients had large hepatic adenomas associated with moderate to severe unremitting microcytic anemia and hypoferremia, unresponsive to oral iron and only partially responsive to parenteral iron [47]. The anemia and hypoferremia rapidly resolved after tumor resection. A sample of tumor tissue overexpressed hepcidin mRNA which was suppressed in the surrounding liver, suggesting autonomous production of hepcidin by the tumor. A patient with a similar presentation in the setting of a large hepatic adenoma but no history of glycogen disorder was recently reported [48].

### 4.4. Iron-Loading Anemias

In iron-loading anemias, such as *β*-thalassemia and congenital dyserythropoietic anemias, urinary and serum hepcidin are severely decreased in the absence of transfusions [49–51]. The low hepcidin in turn allows excessive iron absorption and development of systemic iron overload, similar to hereditary hemochromatosis. The signal causing hepcidin suppression in iron-loading anemias appears to be generated by high erythropoietic activity and outweighs the effects of the resulting iron overload on hepcidin regulation. As mentioned earlier, GDF15 and TWSG1 are two erythroid factors that may contribute to hepcidin suppression in syndromes with ineffective erythropoiesis [32, 33].

Hepcidin diagnostics may be useful for iron-loading anemias to identify the patients at higher risk of iron toxicity due to severely decreased hepcidin levels. Moreover, future hepcidin agonists may be sufficient to prevent the life-threatening iron overload in these patients. The first promising evidence of the beneficial effect of hepcidin comes from the th3/+ mouse model of *β*-thalassemia in which moderate transgenic expression of hepcidin resulted in lower spleen and liver iron content, decreased inefficient erythropoiesis in the spleen, lower spleen weight, and even improvement of hematological parameters (Gardenghi et al., submitted).

In chronically transfused patients, hepcidin concentrations are much higher than in nontransfused patients, presumably due to both increased iron load and the alleviation of ineffective erythropoiesis [49, 51]. Interestingly, Origa et al. [51] showed that nontransfused patients have liver iron concentrations similar to those of regularly transfused thalassemia major patients. However, because of the different hepcidin levels, the cellular distribution of iron in the liver differed in these two groups. In nontransfused thalassemia, iron was deposited in hepatocytes, whereas higher hepcidin levels in transfused patients resulted in macrophage iron loading. As a consequence of this difference in cellular iron distribution, serum ferritin levels were much lower in nontransfused patients, and did not adequately reflect the patients' liver iron load. Considering that high hepcidin shifts iron distribution to macrophages and decreases intestinal iron absorption, it is possible that hepcidin agonists could be useful even in transfused thalassemia patients. Trapping iron in macrophages where it is less toxic may postpone iron deposition and consequent damage in the parenchyma, but this remains to be investigated.

### 4.5. Anemia of Inflammation

Anemia of inflammation (AI) develops in the setting of many infections and inflammatory disorders, and some malignancies [52]. Elevated hepcidin is believed to be an important mediator of AI but whether hepcidin is a necessary factor in the AI pathogenesis has not yet been established with certainty. IRIDA and mouse models overexpressing hepcidin demonstrate that elevated hepcidin is sufficient to cause hypoferremia and anemia [4]. Moderate overproduction of hepcidin in transgenic mice or in mice bearing hepcidin-producing tumors caused an iron-restricted anemia [4, 53]. Transgenic mice also had blunted erythropoietic response to EPO, another characteristic of AI.

In humans, elevated hepcidin was observed in a variety of inflammatory disorders including rheumatologic diseases, inflammatory bowel disease, infections, multiple myeloma, and critical illness [13, 35–37, 54, 55]. The AI phenotype presumably develops from hepcidin-mediated inhibition of iron recycling and absorption. Decreased flow of iron into plasma results in hypoferremia, and because most of the iron in plasma is destined for the bone marrow, lower iron availability hinders hemoglobin synthesis and erythrocyte production. 

Detecting elevated hepcidin in the presence of hypoferremia and anemia could help distinguish AI from IDA. However, mixed anemia is common, for example, in cases of chronic inflammatory illness with coexisting bleeding or malnutrition. In these conditions, the inflammation-mediated increase in hepcidin would be opposed by the effects of iron deficiency. Even without blood loss or malnutrition, when inflammation lasts for years, true iron deficiency may also develop because of the inhibition of intestinal iron absorption by hepcidin. The ability of hepcidin measurements to distinguish the contribution of iron deficiency and inflammation in these conditions will have to be carefully evaluated. It is possible that even “normal” levels of hepcidin in AI or mixed anemia are inappropriate and perpetuate iron restriction. Obesity, itself a chronic inflammatory condition, was reported to be associated with iron deficiency [56], and this can develop despite adequate iron supply and the absence of blood loss. Serum hepcidin concentrations were recently studied in obese premenopausal women [57] and were found to be in the reference range for the healthy iron-replete women. However, when compared to lean volunteers with similar iron deficiency, hepcidin levels were several-fold higher in obese women, indicating they were inappropriately high considering the obese women's iron deficiency. Thus, in very chronic mild inflammatory conditions even a mild hepcidin excess may be sufficient to tip the balance between iron loss and iron uptake and lead to iron deficiency. 

Studies of interventions that selectively reduce hepcidin will clarify how essential the role of hepcidin is in each type of inflammation-induced anemia. In a mouse model of AI caused by injections of Brucella abortus, hepcidin antagonists (neutralizing monoclonal antibody to hepcidin) in combination with erythropoiesis-stimulating agents restored normal hemoglobin levels [58], even when erythropoiesis-stimulating agents alone were ineffective. Considering the variety of other possible inflammatory erythroid pathologies, ranging from hemolysis to bone marrow suppression, not all inflammatory conditions may respond equally to antihepcidin or combination therapies.

### 4.6. Anemia of Chronic Kidney Disease

Until recently, anemia of chronic kidney disease (CKD) was thought to be primarily due to the deficiency of erythropoietin. Considering that high amounts of erythropoietin are often needed to restore erythropoiesis, this view has been challenged. This, as well as the beneficial effect of high doses of intravenous iron in CKD patients has focused attention on the possible role of hepcidin and iron restriction in the anemia of CKD [59]. Renal excretion is a major route of hepcidin clearance. When kidney function is normal, urinary hepcidin concentrations correlate well with the circulating hepcidin levels, with no apparent regulation of the excretion process. However, loss of kidney function could decrease hepcidin clearance and lead to the accumulation of hepcidin and the development of iron-restrictive anemia.

Hepcidin concentrations were indeed reported to be increased in patients with CKD [54, 60]. Although this could be caused by inflammation which frequently accompanies CKD, even patients without significant inflammation had elevated hepcidin which progressively increased with the increasing severity of CKD [60]. Decreased kidney function is the likely factor contributing to this phenomenon, and some studies have reported inverse correlation between glomerular filtration rate and serum hepcidin [60, 61]. Thus, increased hepcidin levels due to decreased renal clearance as well as due to inflammation may be a significant factor contributing to the development of anemia in CKD and should be considered in the development of new therapies for this disease.

### 4.7. Resistance to Erythropoietin

Hyporesponsiveness to therapeutic erythropoietin has emerged as an important consequence of inflammation, especially in chronic kidney diseases [62, 63], and may be a consequence of iron restriction imposed by high hepcidin. As mentioned before, hepcidin-overexpressing transgenic mice had blunted response to Epo [4], and neutralization of hepcidin by monoclonal antibody in the B. abortus model of AI restored responsiveness to erythropoietin [58]. Hepcidin measurements thus may be useful for predicting patients′ response to erythropoietin but large studies will be necessary to test this concept. 

Large pharmacological doses of Epo can sometimes overcome the resistance of AI to erythropoietin [63]. As was discussed before, Epo injections in mice and humans resulted in suppression of hepcidin production [29, 30], and this may be the mechanism by which high Epo levels overcome iron restriction. It is therefore conceivable that administration of anti-hepcidin therapies together with erythropoiesis-stimulating agents may improve patients' erythropoietic response and enable the use of lower erythropoietin doses to avoid the potential detrimental effects of high Epo concentrations. 

## 5. The Present and the Future of Hepcidin Diagnostics and Therapeutics in Anemias

### 5.1. Diagnostics

The evaluation of the diagnostic potential of hepcidin has only recently become possible with the development of assays for bioactive mature hepcidin in serum and urine. The methodologies include competitive ELISAs using biotinylated or radioiodinated hepcidin as tracers [13, 64], and several mass spectrometry-based assays (MALDI-TOF MS, SELDI, and LC–MS/MS) using as internal standards isotopically labeled hepcidin or truncated hepcidin variants [55, 65, 66]. Measurements of prohepcidin in serum have also been reported, but these did not correlate with mature hepcidin concentrations indicating that pro-hepcidin is inadequate as a substitute for measuring biologically relevant hepcidin. 

A summary of potential uses of hepcidin assays for diagnosing different forms of anemia is listed in Table 1. However, the utility of hepcidin for the diagnosis and prognosis of iron disorders is far from understood and needs to be evaluated in larger clinical studies. Some of the problems for interpreting hepcidin levels may include diurnal fluctuations of hepcidin (lower in the morning, higher in the afternoon) [13, 67], and relative sensitivity to the available iron content of the diet [13].

Hepcidin concentrations may also predict which patients could benefit from oral iron therapy. Several studies of healthy volunteers have shown inverse correlation between hepcidin concentrations and radiolabeled iron absorption [68, 69]. If these results are confirmed in appropriate patient populations, patients with elevated hepcidin levels could be treated with parenteral iron and thus avoid weeks of potentially burdensome and unsuccessful oral iron therapy.

### 5.2. Therapeutics

The potential use of hepcidin-targeted therapeutics in anemias is summarized in Table 1. Although no hepcidin therapies are yet available, several candidates are currently under development. Hepcidin agonists would be useful for preventing or ameliorating iron overload in nontransfused *β*-thalassemias and other iron-loading anemias. Small peptides based on hepcidin N-terminal region have been shown to act as agonists in mice in vivo (E. Nemeth, unpublished). The small size should allow eventual development of orally available agonists. 

Hepcidin antagonists would be expected to benefit patients with diseases of hepcidin excess (Table 1), manifested as iron-restricted anemia and eventually as systemic iron deficiency. Several approaches have been undertaken to develop hepcidin antagonists. Hepcidin-neutralizing antibody has already been successfully used in vivo in a mouse model of AI [58]. Apart from directly interfering with hepcidin activity, other agents which target pathways regulating hepcidin production have also been described. Dorsomorphin, a small-molecule inhibitor of BMP signaling, was shown to block hepcidin induction by iron in vivo [70]. Soluble HJV, also acting as an antagonist of BMP signaling, decreased hepcidin baseline expression in mice and concurrently increased liver iron content [71]. Furthermore, some existing therapies may be acting in part by decreasing hepcidin production. Anticytokine therapies such as anti-IL-6 antibody were shown to suppress hepcidin production and improve anemia [72, 73]. Some erythropoiesis-stimulating agents, such as prolyl hydroxylase inhibitors, could also be effective hepcidin suppressors, not only by stimulating erythropoiesis, but also by interfering with the HIF pathway [74]. Undoubtedly, future studies will assess the risks and relative benefits of hepcidin-targeted treatment approaches.

## 6. Conclusion

Recent advances in the understanding of the key role of hepcidin-ferroportin interaction in iron homeostasis and its disorders have clarified the pathogenesis of anemias due to iron restriction as well as anemias accompanied by iron-loading. While many mechanistic details of hepcidin and ferroportin regulation remain to be worked out, we will soon see medical applications of these advances. In the coming years, the role of hepcidin assays in the diagnosis, prognosis, and therapeutic stratification of anemias will be explored. Therapeutic interventions specifically targeting the hepcidin-ferroportin axis for the treatment of anemias are also under development.

## Figures and Tables

**Table 1:** Diagnostic and therapeutic potenital of hepcidin in different forms of anemia.

Condition	Expected hepcidin levels	Other iron parameters	Hepcidin therapy
Iron deficiency anemia	Low	Low Tsat and ferritin	
Iron-refractory iron deficiency anemia	High	Low Tsat and ferritin	Antagonist
Iron-loading anemias	Low (unless transfused)	High Tsat and ferritin	Agonist
Anemia of inflammation	High	Low Tsat, normal-to-elevated ferritin	Antagonist
Mixed anemia (AI/IDA)	Normal	Low Tsat, low-to-normal ferritin	Antagonist
Chronic kidney disease	High	Variable	Antagonist
Erythropoietin resistance	High	Variable	Antagonist

Tsat = transferrin saturation.
